# A Longitudinal Analysis of SARS-CoV-2 Antibody Responses Among People With HIV

**DOI:** 10.3389/fmed.2022.768138

**Published:** 2022-03-07

**Authors:** Maria L. Alcaide, Nicholas F. Nogueira, Ana S. Salazar, Emily K. Montgomerie, Violeta J. Rodriguez, Patricia D. Raccamarich, Irma T. Barreto, Angela McGaugh, Mark E. Sharkey, Alejandro M. Mantero, Allan E. Rodriguez, Laura Beauchamps, Deborah L. Jones

**Affiliations:** ^1^Division of Infectious Diseases, Department of Medicine, University of Miami Miller School of Medicine, Miami, FL, United States; ^2^Department of Psychiatry and Behavioral Sciences, University of Miami Miller School of Medicine, Miami, FL, United States; ^3^Department of Medicine, University of Miami Miller School of Medicine, Miami, FL, United States; ^4^Department of Public Health Sciences, University of Miami Miller School of Medicine, Miami, FL, United States

**Keywords:** antibody, SARS-CoV-2, HIV, adaptive immunity, innate immunity, COVID-19 vaccine

## Abstract

**Background:**

The concentration and duration of antibodies (Ab) to SARS-CoV-2 infection predicts the severity of the disease and the clinical outcomes. Older people and those with HIV have impaired immune responses, worse outcomes after SARS-CoV-2 infection, and lower antibody responses after viral infection and vaccination. This study evaluated an Ab response to SARS-CoV-2 in people with HIV (PWH) and without HIV (HIV-) and its association with age.

**Methods:**

A total of 23 COVID+PWH and 21 COVID+HIV- participants were followed longitudinally for 6 months post-mild COVID-19. Immunoglobin G (IgG) and immunoglobin M (IgM) Ab responses were measured by an in-house developed ELISA. Time points and HIV status interaction were analyzed using Poisson generalized estimating equations, and correlations were analyzed using non-parametric tests.

**Results:**

Median age in PWH was 55 years with 28.6% women, while in the HIV- group was 36 years with 60.9% women. The mean time from COVID-19 diagnosis to study enrollment was 16 days for PWH and 11 days for HIV-. The mean CD4+ T-cell count/μl for PWH was 772.10 (±365.21). SARS-CoV-2 IgM and IgG were detected at all time points and Ab response levels did not differ by HIV status (*p* > 0.05). At entry, age showed a weak direct association with IgG responses (ρ = 0.44, *p* < 0.05) in HIV- but did not show any association in PWH. Similar associations between age, IgG, and HIV status emerged at day 14 (T1; ρ = 0.50, *p* < 0.05), 3 months (T3; ρ = 0.50, *p* < 0.05), and 6 months visit (T4; ρ = 0.78, *p* < 0.05) in the HIV- group.

**Conclusion:**

The Ab responses in the 6-month post-SARS-CoV-2 infection did not differ by HIV status, though a positive association was found between age and Ab response in older PWH. Results suggest that immune protection and vaccine responses are similar for PWH than for those without HIV infection.

## Introduction

Coronavirus Disease 2019 (COVID-19) has caused high mortality and morbidity worldwide since it appeared in December 2019 (1, 2). The severity and progression of COVID-19 vary among individuals and appear to be linked to the role of the host immunity and adequate antibody (Ab) responses (3–5). While data suggests that after natural infection of SARS-CoV-2, the development of anti-spike protein immunoglobulin M (IgM) and immunoglobulin G (IgG) occurs at approximately 10 days after the onset of symptoms, (6–9) the longevity of Abs mediated by adaptive immunity to SARS-CoV-2 has not been fully determined (10–13).

Ab targeting viral surface glycoproteins mediate humoral immune responses to SARS-CoV-2 (14). The 180 kDa spike glycoprotein is considered an important antigenic determinant capable of inducing a protective immune response (15). The S1 subunit is the receptor-binding domain (RBD), which mediates a viral binding to the functional ACE2 receptors on susceptible cells and is the main target for SARS-CoV-2 neutralizing Ab (14). The Ab against the RBD region has been considered a reliable indicator of recent severe SARS-CoV-2 infection, future seroprotection, and vaccine response.

Several studies have described higher rates of COVID-19-related diagnosis, hospitalizations, and mortality in people with HIV (PWH) compared to those without HIV (HIV-) (16, 17). While root causes of poor outcomes remain uncertain, persistent state of immune activation and inflammation have been suggested as plausible reasons for worse outcomes in PWH (18–21). Furthermore, recent studies have found that SARS-CoV-2-coinfected PWH are almost 10 years younger than those who are HIV- despite having similar underlying medical conditions (21, 22). The difference may be related to the fact that HIV accentuates the biological age of those infected individuals, which could have important health implications when establishing a high-risk threshold for age for the population (21, 23).

Understanding the host immune response and duration of Ab levels after SARS-CoV-2 is crucial for planning for healthcare and public health interventions. Thus, in this study, we aimed to evaluate the Ab response to SARS-CoV-2 of PWH and those who are HIV- and its association with age.

## Materials and Methods

### Participants

We recruited participants from the community as part of the ongoing A Comprehensive Translational Initiative On Novel coronavirus (ACTION) Cohort, evaluating SARS-CoV-2 coinfection in Miami. Participants were identified through an existing patient registry at the Miami Center for HIV Research in Mental Health (CHARM) and Center for AIDS Research (CFAR). In addition, participants were recruited *via* medical referrals, word-of-mouth, and/or recruitment flyers.

Inclusion criteria for the study were individuals ≥18 years in age with a documented diagnosis of SARS-CoV-2 infection by a commercially approved PCR test within 4 weeks. All participants had mild COVID-19 symptoms without hospitalization. PWH were on effective antiretroviral therapy (ART; plasma viral load <500 copies/ml) as determined by recent HIV viral load. Participants attended in-person visits between May 2020 and March 2021 and were followed longitudinally at baseline (T0), day 14 (T1), 1 month (T2), 3 months (T3), and 6 months (T4). Following screening and enrollment, a Food and Drug Authority (FDA) Emergency Use Authorized IgG/IgM rapid test was performed (24).

### Clinical Assessments

Eligible participants completed a 20-min phone questionnaire. The questionnaire is an adaptation from the survey in the Multicenter AIDS Cohort Study/Women's Interagency HIV Study (MACS/WIHS) Combined Cohort Study (MWCCS) previously used to evaluate a COVID-19 burden among PWH in the U.S. and other settings (25–27). This questionnaire collected sociodemographic information (i.e., sex, gender, race, ethnicity, employment status, living situation, and monthly household income), clinical data on COVID-19 exposure risk (i.e., recent travel, healthcare worker, use of public transportation, and contact with infected persons), clinical presentation, medical history, and comorbidities.

### Laboratory Assessments: Spike RBD IgG and IgM ELISA

Spike RBD antigen-specific IgG and IgM levels were measured by a quantitative ELISA as described by Krammer lab (28). Briefly, 96 well-plates were coated with 1 μg/ml SARS-CoV-2 antigen overnight at 4°C, followed by blocking with 3% skim milk in PBS containing 0.05% Tween-20 for 2 h at room temperature. Heat inactivated plasma was added to the plates at 1:100 dilution and incubated at room temperature followed by washing and addition of anti-human IgG peroxidase or anti-human IgM peroxidase secondary Ab. After incubation, plates were developed with 3,3',5,5'-Tetramethylbenzidine (TMB) substrate and followed by a stopping reaction with 2M sulfuric acid. The plates were read using an ELISA plate reader at 450 nm and optical densities were background subtracted. A positive control standard was created by pooling the plasma from eight patients with convalescing COVID-19. The positive control standard was run on each plate and was used to calculate titers (relative units) for all samples using non-linear regression interpolations to quantify the amount of anti-RBD IgG and anti-RBD IgM present in each specimen. The cut-off was calculated based on the mean titer for the negative control +3 SD above the mean. The COVID-19 negative controls were samples from HIV uninfected individuals collected in 2018 (prior to the identification of SARS-COV-2 and the COVID-19 pandemic) under other IRB-approved protocols. Laboratory assessments were conducted in the University of Miami CFAR laboratories.

### Statistical Analysis

Data and statistical analyses were conducted using SPSS version 24. The data plotted were expressed as mean ± SD or median values. Poisson generalized estimating equations were used to analyze time points, HIV status, and their interactions to predict Ab response. Correlation analyses were performed using non-parametric correlations (Spearman). A *p* < 0.05 was considered significant.

## Results

### Sample Characteristics

The sociodemographic characteristics, COVID-19 risk exposure, clinical presentation, and comorbidities are illustrated in Table 1. A total of 44 participants were enrolled in the study, of which 23 were PWH and 21 were HIV-. The median age of all participants was 48 years (range 22–78) with 45.5% women. The median age for PWH was 55 years (range 26–68) with 28.6% women, and HIV- was 36 years (range 26–68) with 60.9% women. Most participants self-identified as White (Overall: 64.1%; PWH: 52.4%; HIV-: 69.6%) and Hispanic (Overall: 84.1%, PWH: 81%, HIV-: 87%). Almost half were employed (52.3% overall). The most common risk exposure was suspected contact with a person with a COVID-19 diagnosis.

**Table 1:** Sociodemographic, exposure risk, symptoms, and comorbidities of 44 individuals with SARS-CoV-2 by HIV status.

	**Overall (*N* = 44)**	**PWH (*N* = 23)**	**HIV (-) (*N* = 21)**
Median age in years (Min, Max)	48 (22, 78)	55 (26, 68)	36 (22, 78)
**Race**			
Black	6 (13.6)	5 (23.8)	1 (4.3)
White	27 (61.4)	11 (52.4)	16 (69.6)
Native American	1 (2.3)	0 (0.0)	1 (4.3)
Other	10 (22.7)	5 (23.8)	5 (21.7)
**Ethnicity**			
Hispanic	37 (84.1)	17 (81.0)	20 (87.0)
Non-Hispanic	4 (9.1)	2 (9.5)	2 (8.7)
Other	3 (6.8)	2 (9.5)	1 (4.3)
Female	20 (45.5)	6 (28.6)	14 (60.9)
Full/part-time employment	23 (52.3)	8 (38.1)	15 (65.2)
**Exposure risk**			
Healthcare worker	10 (22.7)	2 (9.5)	8 (34.8)
Public transportation use	11 (25.0)	8 (38.1)	3 (13.0)
Suspected contact with person with COVID-19	28 (63.6)	12 (57.1)	16 (69.6)
Absolute CD4+ T-cell count/μl, mean (± SD)	–	772.10 (365.21)	–
**SARS-CoV-2 diagnosis**			
Positive rapid test result at screening	42 (97.7)	20 (95.2)	22 (100.0)
Mean time from positive PCR in days (± SD)	15.11 (12.99)	15.93 (13.85)	11.00 (7.81)
**Symptoms**			
Fever/elevated temperature	24 (54.5)	8 (38.1)	12 (52.2)
Chills	22 (50.0)	13 (61.9)	9 (39.1)
Muscle aches/myalgia	35 (79.5)	13 (61.9)	22 (95.7)
Eye redness/conjunctivitis	7 (15.9)	4 (19.0)	3 (13.0)
Upper respiratory tract^*^	16 (36.4)	10 (47.6)	6 (26.1)
Dyspnea	11 (25.0)	5 (23.8)	6 (26.1)
Confusion	9 (20.5)	5 (23.8)	4 (17.4)
Headache	32 (72.7)	19 (82.6)	13 (61.9)
Loss of taste/ageusia	31 (70.5)	14 (66.7)	17 (73.9)
Loss of smell/anosmia	32 (72.7)	15 (71.4)	17 (73.9)
Gastrointestinal symptoms^**^	12 (27.3)	5 (23.8)	7 (30.4)
Concurrent symptoms	34 (77.3)	14 (66.7)	20 (87.0)
**Comorbidities**			
Cardiovascular disease risk^***^	1 (2.3)	0 (0.0)	1 (4.3)
Active malignancy	3 (6.8)	2 (9.5)	1 (4.3)
Pulmonary disease	5 (11.4)	4 (19.0)	1 (4.3)
Rheumatological disease	2 (4.5)	0 (0.0)	2 (8.7)

* *Upper Respiratory Tract Symptoms include runny nose, sore throat, or cough*.

* *Gastrointestinal Symptoms include Nausea/vomiting, diarrhea, or abdominal pain.

*** *Cardiovascular Disease Risk includes Hypertension, Heart Disease, Diabetes Mellitus, and renal disease*.

*Data as presented as No. (%) unless otherwise indicated*.

Mean time from COVID-19 diagnosis to enrollment was 16 days for PWH and 11 days for HIV-. The mean CD4+ T-cell count/μl for the PWH was 772.10 (± 365.21). The prevailing clinical presentations reported were as follows in decreasing order of frequency: myalgia, headache, loss of smell, loss of taste, fever/elevated temperature, chills, upper respiratory tract symptoms, gastrointestinal symptoms, dyspnea, confusion, and eye redness/conjunctivitis. Over three-fourths had one or more symptoms concurrently. The most frequently reported comorbidity was cardiovascular disease risks factors (hypertension, heart disease, diabetes mellitus, and renal disease). History of pulmonary disease (asthma and bronchitis), active malignancy, or rheumatological diseases was uncommon.

### Antibody Responses to SARS-CoV-2

**Figure 1:**
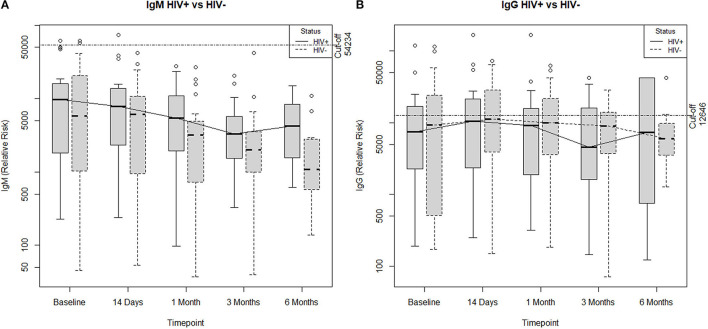
Poisson generalized estimated equations comparing **(A)** IgM antibody response and **(B)** IgG antibody response across all time points by HIV status. Antibody responses in the 6-month post-SARS-CoV-2 infection did not differ by HIV status.

Ab responses to SARS-CoV-2 by HIV status can be found in Table 2. The IgM and IgG Ab responses specific to RBD antigen were analyzed at baseline (T0), day 14 (T1), 1 month (T2), 3 months (T3), and 6 months (T4) post-study entry. PWH showed similar levels of RBD-specific IgM and IgG compared to HIV- individuals post-CoV2 infection at all time points. Overall, the data indicate that levels and persistence of Ab response to SARS-CoV2 infection in PWH are comparable to the HIV- population (Figure 1).

**Table 2:** Time points predicting immunoglobin G (IgG) and immunoglobin M (IgM) to SARS-CoV-2 by HIV status.

**Variables**	**IgG Levels**			**IgM Levels**		
	***B***	***SE***	***p***	***B***	***SE***	***p***
Intercept^*^	9.33	0.33	<0.01	7.80	0.34	<0.01
T0	0.67	0.48	0.16	1.85	0.40	<0.01
T1	0.64	0.44	0.15	1.35	0.38	<0.01
T2	0.38	0.43	0.38	0.76	0.38	0.04
T3	−0.18	0.37	0.62	0.62	0.59	0.29
HIV status	0.59	0.46	0.21	0.82	0.45	0.06
T0 x HIV-	−0.95	0.61	0.12	−0.84	0.51	0.10
T1 x HIV-	−0.53	0.59	0.37	−0.41	0.48	0.40
T2 x HIV-	−0.51	0.59	0.39	−0.44	0.47	0.35
T3 x HIV-	−0.45	0.50	0.36	−0.73	0.70	0.30

*SE, Standard Error. T0 (baseline) N = 44; T1 (14 days) N = 40; T2 (30 days) N = 42; T3 (90 days) N = 40; T4 (180 days) N = 22*.

* *Reference category for time points is T4 and for HIV status is PWH*.

### Direct Association Between Age and Persistence of SARS-CoV-2 Antibody Responses in PWH

**Figure 2:**
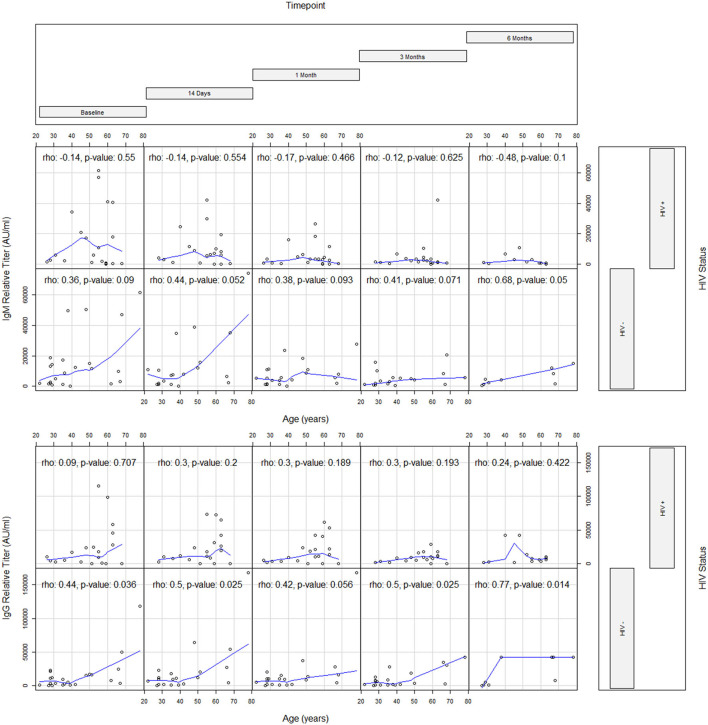
Coplots evaluating the correlation between age and antibody response by timepoint and HIV status.

The association between age and persistence of SARS-CoV-2 Ab responses by HIV status is presented in Table 3. At entry, age showed a weak direct association with IgG responses (ρ = 0.44) in HIV- but did not show any association in PWH. Similar associations between age, IgG, and HIV status emerged at T1 (ρ = 0.50), T3 (ρ = 0.50), and T4 (ρ = 0.78) (Table 3 and Figure 2). At T4, an association between age and IgM also emerged (ρ = 0.68) in the HIV- group. Additionally, absolute CD4+ T cell count in PWH did not correlate with IgM and IgG responses.

**Table 3:** Correlation matrix for age, IgG, and IgM at each time point.

			**Status**			
	**Overall**		**PWH**		**HIV (-)**	
**Visit (days)**	**IgG**	**IgM**	**IgG**	**IgM**	**IgG**	**IgM**
T0 (0)	0.27	0.14	0.09	−0.14	0.44^*^	0.36
T1 (14)	0.39^**^	0.09	0.30	−0.14	0.50^*^	0.44
T2 (30)	0.37	0.06	0.30	−0.17	0.42	0.38
T3 (90)	0.45^**^	0.13	0.30	−0.12	0.50^*^	0.41
T4 (180)	0.60^**^	0.16	0.24	−0.48	0.78^*^	0.68^*^

*Spearman correlations reported. T0 N = 44; T1 N = 40; T2 N = 42; T3 N = 40; T4 N = 22*.

* *p <0.05 (two-tailed)*.

** *p <0.01 (two-tailed)*.

## Discussion

This study compared the magnitude and longevity of SARS-CoV-2 Ab responses in a group of virologically suppressed PWH and HIV- controls after mild COVID-19. It also evaluated the relationship of SARS-CoV-2 Ab responses and age by HIV status. While it was hypothesized that an impaired immune response would result in lower and shorter term Ab responses in PWH, our results show that PWH can develop appropriate Ab responses to SARS-CoV-2.

These results support the growing literature on similar magnitude and durability of Ab titers in individuals with and without HIV after natural infections (29–31). In addition, similar early and acute Ab responses and time to peak Ab titer of anti-SARS-CoV-2 IgM and IgG have been observed between these groups, which are considered a marker of increased protection (29). Our findings, however, contrast with those of Spinelli et al. (34), as our results indicate that the level of Ab responses to SARS-CoV2 infection in PWH is comparable to those without HIV infection. These discrepancies may be related to the different characteristics of the study participants, such as the lower number of comorbidities, higher CD4+ T-cell count, and an HIV viral suppression seen in our participants. These findings could shed light on the understanding and management of HIV in the era of COVID-19 and highlight the importance of ensuring appropriate ART adherence during the pandemic.

Previous literature has found impaired immune responses occurring in the setting of HIV and aging (32–34). Immune responses affected by aging can lead to reduced or delayed Ab responses through cell-intrinsic defects, functional defects of T-cells, and antigen uptake, resulting in worse outcomes compared to those uninfected (35). While it is thought that HIV infection adds several years to the chronological age of those infected (21, 23) even with a higher median age of 19 years, PWH in this study had comparable Ab responses to the younger HIV-individuals. On the other hand, the HIV- group was younger than the HIV infected group, which could partially explain the lack of association between age and SARS-CoV-2 Ab titers. Recent data, however, showed that participants aged 19–30 years had significantly lower IgG levels than those aged 50–80 but not those aged 81 or older (36).

Among the general population, older age and comorbidities, including immunosuppression, are linked to severe outcomes and death due to SARS-CoV-2 (37). The limited data on PWH coinfected with SARS-CoV-2 suggest that PWH with adequate immune recovery and viral suppression have similar or better clinical course than those without HIV infection when developing COVID-19 (38, 39). For instance, a case-series report denoted similar clinical and immunological data on virally suppressed PWH hospitalized patients who showed high degrees of both cytokines production and immune activation (40). Despite having a mild presentation, IgG production was elicited in all patients and neutralized the Ab in all but one patient.

Because SARS–CoV-2 infection is associated with a broad spectrum of disease, heterogeneous Ab responses are expected. The SARS-CoV-2 Ab testing has an important role in providing insights about a previous infection, which is important for community surveillance and for determining short-term immunity and protection against reacquiring infection. As many PWHs are likely at high risk of becoming infected with SARS-CoV-2 and severe COVID-19 due to comorbid conditions (41, 42), the development of IgM and IgG Ab against the spike protein could likely indicate protection against reinfections and potential protection after vaccination against SARS-CoV-2. As such, larger and more diverse studies are needed to understand the host immune response and duration of Ab levels after SARS-CoV-2 in the HIV setting.

Our study is limited by the small sample size, a short longitudinal follow-up, and evaluation of patients at different time points after infection based on positive PCR results, which may have resulted in the participant recall bias when describing clinical presentation, and in differences in Ab titers. Due to a lack of data on antibody responses at the time the study was conducted, no *a priori* power analysis was conducted to determine our sample size. This study did not include participants who received a COVID-19 vaccine, had a history of hospitalization for severe disease or had a low CD4+ T-cell count as most PWH in our community are on an effective ART. This is a neoteric immunological study in co-infection of PWH and SARS-CoV-2, providing insights into short-term immunity and safeguarding against re-infection - critical for designing public health initiatives.

In summary, this study provides initial information about the magnitude and longevity of Ab responses to SARS-CoV-2 among PWH who developed mild COVID-19. Although extended longitudinal follow-ups are needed, results from this study are promising for PWH. Further characterization of these immunological dynamics might predict protection after SARS-CoV-2 vaccination.

## Data Availability Statement

The data underlying this article will be shared upon reasonable request to the corresponding author.

## Ethics Statement

The study was approved by the University of Miami Institutional Review Board (IRB# 20200340) and informed consent was obtained prior to study assessments. All procedures were followed in accordance with the ethical standards of the University and with the Helsinki Declaration of 1975, as revised in 2013.

## Author Contributions

MA and DJ were responsible for the study concept and design. AS, NN, AM, and VJR contributed with the data analyses. All the authors had access to the full data and were responsible for drafting and critical revision of the manuscript for important intellectual content, they were also responsible for the accuracy and integrity of the data analysis, and for data acquisition and interpretation of results.

## Funding

This study was supported by National Institutes of Health grants to the University of Miami Center for AIDS Research grant [P30A1073961 to MA] and the Center for HIV and Research in Mental Health [P30MH116867 to DJ]. VJR's work on this study was partially supported by a Ford Foundation Fellowship, administered by the National Academies of Science, a PEO Scholar Award from the PEO Sisterhood, and a grant from the NIH [R36MH127838].

## Conflict of Interest

The authors declare that the research was conducted in the absence of any commercial or financial relationships that could be construed as a potential conflict of interest.

## Publisher's Note

All claims expressed in this article are solely those of the authors and do not necessarily represent those of their affiliated organizations, or those of the publisher, the editors and the reviewers. Any product that may be evaluated in this article, or claim that may be made by its manufacturer, is not guaranteed or endorsed by the publisher.

## References

[1] Zhu N Zhang D Wang W Li X Yang B Song J. A novel coronavirus from patients with pneumonia in China, 2019. N Engl J Med. (2020) 382:727–33. 10.1056/NEJMoa2001017 31978945 PMC7092803

[2] Chen N Zhou M Dong X Qu J Gong F Han Y. Epidemiological and clinical characteristics of 99 cases of 2019 novel coronavirus pneumonia in Wuhan, China: a descriptive study. Lancet. (2020) 395:507–13. 10.1016/S0140-6736(20)30211-7 32007143 PMC7135076

[3] Zohar T Alter G. Dissecting antibody-mediated protection against SARS-CoV-2. Nat Rev Immunol. (2020) 20:392–4. 10.1038/s41577-020-0359-5 32514035 PMC7278217

[4] Samadizadeh S Masoudi M Rastegar M Salimi V Shahbaz MB Tahamtan A. COVID-19: why does disease severity vary among individuals? Respir Med. (2021) 180:106356. 10.1016/j.rmed.2021.106356 33713961 PMC7934673

[5] Gervasoni C Meraviglia P Riva A Giacomelli A Oreni L Minisci D. Clinical features and outcomes of HIV patients with coronavirus disease 2019. Clin Infect Dis. (2020) 71:2276–8. 10.1093/cid/ciaa579 32407467 PMC7239244

[6] Wang B Wang L Kong X Geng J Xiao D Ma C. Long-term coexistence of SARS-CoV-2 with antibody response in COVID-19 patients. J Med Virol. (2020) 92:1684–9. 10.1002/jmv.25946 32343415 PMC7267623

[7] Koblischke M Traugott MT Medits I Spitzer FS Zoufaly A Weseslindtner L. Dynamics of CD4 T Cell and antibody responses in COVID-19 patients with different disease severity. Front Med. (2020) 7:592629. 10.3389/fmed.2020.592629 33262993 PMC7686651

[8] Jiang XL Wang GL Zhao XN Yan FH Yao L Kou ZQ. Lasting antibody and T cell responses to SARS-CoV-2 in COVID-19 patients three months after infection. Nat Commun. (2021) 12:897. 10.1038/s41467-021-21155-x 33563974 PMC7873066

[9] Chen Y Zuiani A Fischinger S Mullur J Atyeo C Travers M. Quick COVID-19 healers sustain anti-SARS-CoV-2 antibody production. Cell. (2020) 183:1496–507.e16. 10.1016/j.cell.2020.10.051 33171099 PMC7608032

[10] Thevarajan I Nguyen THO Koutsakos M Druce J Caly L van de Sandt CE. Breadth of concomitant immune responses prior to patient recovery: a case report of non-severe COVID-19. Nat Med. (2020) 26:453–5. 10.1038/s41591-020-0819-2 32284614 PMC7095036

[11] Long QX Liu BZ Deng HJ Wu GC Deng K Chen YK. Antibody responses to SARS-CoV-2 in patients with COVID-19. Nat Med. (2020) 26:845–8. 10.1038/s41591-020-0897-1 32350462

[12] Hou H Wang T Zhang B Luo Y Mao L Wang F. Detection of IgM and IgG antibodies in patients with coronavirus disease 2019. Clin Transl Immunology. (2020) 9:e01136. 10.1002/cti2.1136 32382418 PMC7202656

[13] Gandhi RT Lynch JB Del Rio C. Mild or moderate Covid-19. N Engl J Med. (2020) 383:1757–66. 10.1056/NEJMcp2009249 32329974

[14] Piccoli L Park YJ Tortorici MA Czudnochowski N Walls AC Beltramello M. Mapping neutralizing and immunodominant sites on the SARS-CoV-2 spike receptor-binding domain by structure-guided high-resolution serology. Cell. (2020) 183:1024–42.e21. 10.1016/j.cell.2020.09.037 32991844 PMC7494283

[15] Daniel C Talbot PJ. Protection from lethal coronavirus infection by affinity-purified spike glycoprotein of murine hepatitis virus, strain A59. Virology. (1990) 174:87–94. 10.1016/0042-6822(90)90057-X 2152996 PMC7131235

[16] Bhaskaran K Rentsch CT MacKenna B Schultze A Mehrkar A Bates CJ. HIV infection and COVID-19 death: a population-based cohort analysis of UK primary care data and linked national death registrations within the OpenSAFELY platform. Lancet HIV. (2020) 8:e24–32. 10.1101/2020.08.07.20169490 33316211 PMC7773630

[17] Castel AD Wilbourn B Magnus M Greenberg AE. SARS-CoV-2 and HIV: epidemiology, treatment, and lessons learned from HIV. AIDS Rev. (2020) 22:133–42. 10.24875/AIDSRev.20000070 33118529

[18] Ssentongo P Heilbrunn ES Ssentongo AE Advani S Chinchilli VM Nunez JJ. Epidemiology and outcomes of COVID-19 in HIV-infected individuals: a systematic review and meta-analysis. Sci Rep. (2021) 11:6283. 10.1038/s41598-021-85359-3 33737527 PMC7973415

[19] Shiau S Krause KD Valera P Swaminathan S Halkitis PN. The burden of COVID-19 in people living with HIV: a syndemic perspective. AIDS Behav. (2020) 24:2244–9. 10.1007/s10461-020-02871-9 32303925 PMC7165075

[20] Ambrosioni J Blanco JL Reyes-Urueña JM Davies M-A Sued O Marcos MA. Overview of SARS-CoV-2 infection in adults living with HIV. Lancet HIV. (2021) 8:e294–305. 10.1016/S2352-3018(21)00070-9 33915101 PMC8075775

[21] Prabhu S Poongulali S Kumarasamy N. Impact of COVID-19 on people living with HIV: a review. J Virus Erad. (2020) 6:100019. 10.1016/j.jve.2020.100019 33083001 PMC7560116

[22] Vizcarra P Perez-Elias MJ Quereda C Moreno A Vivancos MJ Dronda F. Description of COVID-19 in HIV-infected individuals: a single-centre, prospective cohort. Lancet HIV. (2020) 7:e554–e64. 10.1016/S2352-3018(20)30164-8 32473657 PMC7255735

[23] De Francesco D Wit FW Bürkle A Oehlke S Kootstra NA Winston A. et al. Do people living with HIV experience greater age advancement than their HIV-negative counterparts? AIDS. (2019) 33:259–68. 10.1097/QAD.0000000000002063 30325781 PMC6319574

[24] Caturegli G Materi J Howard BM Caturegli P. Clinical validity of serum antibodies to SARS-CoV-2 : a case-control study. Ann Intern Med. (2020) 173:614–22. 10.7326/M20-2889 32628534 PMC7370852

[25] Jones DL Ballivian J Rodriguez VJ Uribe C Cecchini D Salazar AS. Mental health, coping, and social support among people living with HIV in the Americas: a comparative study between Argentina and the USA during the SARS-CoV-2 pandemic. AIDS Behav. (2021) 25:2391–9. 10.1007/s10461-021-03201-3 33630198 PMC7905200

[26] D'Souza G Springer G Gustafson D Kassaye S Alcaide ML Ramirez C. COVID-19 symptoms and SARS-CoV-2 infection among people living with HIV in the US: the MACS/WIHS combined cohort study. HIV Res Clin Pract. (2020) 21:130–9. 10.1080/25787489.2020.1844521 33211636 PMC7682380

[27] Ballivian J Alcaide ML Cecchini D Jones DL Abbamonte JM Cassetti I. Impact of COVID-19-Related stress and lockdown on mental health among people living with HIV in Argentina. J Acquir Immune Defic Syndr. (2020) 85:475–82. 10.1097/QAI.0000000000002493 33136748

[28] Stadlbauer D Amanat F Chromikova V Jiang K Strohmeier S Arunkumar GA. SARS-CoV-2 seroconversion in humans: a detailed protocol for a serological assay, antigen production, and test setup. Curr Protoc Microbiol. (2020) 57:e100. 10.1002/cpmc.100 32302069 PMC7235504

[29] Snyman J Hwa S-H Krause R Muema D Reddy T Ganga Y. Similar antibody responses against severe acute respiratory Syndrome Coronavirus 2 in individuals living without and with human immunodeficiency virus on antiretroviral therapy during the first South African Infection Wave. Clin Infect Dis. (2021) 73:361–563. 10.1093/cid/ciab758 34472583 PMC8522359

[30] Yamamoto S Saito M Nagai E Toriuchi K Nagai H Yotsuyanagi H. Antibody response to SARS-CoV-2 in people living with HIV. J Microbiol Immunol Infect. (2021) 54:144–6. 10.1016/j.jmii.2020.09.005 33046418 PMC7531336

[31] Alrubayyi A Gea-Mallorquí E Touizer E Hameiri-Bowen D Kopycinski J Charlton B. Characterization of humoral and SARS-CoV-2 specific T cell responses in people living with HIV. Nature Commun. (2021) 12:5839. 10.1101/2021.02.15.431215 34611163 PMC8492866

[32] Pallikkuth S De Armas LR Pahwa R Rinaldi S George VK Sanchez CM. Impact of aging and HIV infection on serologic response to seasonal influenza vaccination. Aids. (2018) 32:1085–94. 10.1097/QAD.0000000000001774 29424779 PMC6574117

[33] George VK Pallikkuth S Parmigiani A Alcaide M Fischl M Arheart KL. HIV infection worsens age-associated defects in antibody responses to influenza vaccine. J Infect Dis. (2015) 211:1959–68. 10.1093/infdis/jiu840 25556252 PMC4836723

[34] Spinelli MA Lynch KL Yun C Glidden DV Peluso MJ Henrich TJ. SARS-CoV-2 seroprevalence, and IgG concentration and pseudovirus neutralising antibody titres after infection, compared by HIV status: a matched case-control observational study. Lancet HIV. (2021) 8:e334–41. 10.1016/S2352-3018(21)00072-2 33933189 PMC8084354

[35] Pinti M Appay V Campisi J Frasca D Fülöp T Sauce D. Aging of the immune system: focus on inflammation and vaccination. Eur J Immunol. (2016) 46:2286–301. 10.1002/eji.201546178 27595500 PMC5156481

[36] Yang HS Costa V Racine-Brzostek SE Acker KP Yee J Chen Z. Association of age with SARS-CoV-2 antibody response. JAMA Netw Open. (2021) 4:e214302. 10.1001/jamanetworkopen.2021.4302 33749770 PMC7985726

[37] Williamson EJ Walker AJ Bhaskaran K Bacon S Bates C Morton CE. Factors associated with COVID-19-related death using openSAFELY. Nature. (2020) 584:430–6. 10.1038/s41586-020-2521-4 32640463 PMC7611074

[38] Mohammed AH Blebil A Dujaili J Rasool-Hassan BA. The risk and impact of COVID-19 pandemic on immunosuppressed patients: cancer, HIV, and solid organ transplant recipients. AIDS Rev. (2020) 22:151–7. 10.24875/AIDSRev.20000052 33118527

[39] Del Amo J Polo R Moreno S Diaz A Martinez E Arribas JR. Incidence and severity of COVID-19 in HIV-positive persons receiving antiretroviral therapy : a cohort study. Ann Intern Med. (2020) 173:536–41. 10.7326/M20-3689 32589451 PMC7394316

[40] Mondi A Cimini E Colavita F Cicalini S Pinnetti C Matusali G. COVID-19 in people living with HIV: clinical implications of dynamics of the immune response to SARS-CoV-2. J Med Virol. (2021) 93:1796–1804. 10.1002/jmv.26556 32975842 PMC7537181

[41] Cooper TJ Woodward BL Alom S Harky A. Coronavirus disease 2019 (COVID-19) outcomes in HIV/AIDS patients: a systematic review. HIV Med. (2020) 21:567–77. 10.1111/hiv.12911 32671970 PMC7405326

[42] Mascolo S Romanelli A Carleo MA Esposito V. Could HIV infection alter the clinical course of SARS-CoV-2 infection? When less is better. J Med Virol. (2020) 92:1777–8. 10.1002/jmv.25881 32293709 PMC7262314

